# Inhibition of HIV Env binding to cellular receptors by monoclonal antibody 2G12 as probed by Fc-tagged gp120

**DOI:** 10.1186/1742-4690-3-39

**Published:** 2006-07-03

**Authors:** James M Binley, Stacie Ngo-Abdalla, Penny Moore, Michael Bobardt, Udayan Chatterji, Philippe Gallay, Dennis R Burton, Ian A Wilson, John H Elder, Aymeric de Parseval

**Affiliations:** 1 Torrey Pines Institute for Molecular Studies, 3550 General Atomics Court, San Diego CA 92121, USA; 2 Department of Molecular Biology, The Scripps Research Institute, 10550 North Torrey Pines Rd. La Jolla, CA 92037, USA; 3 National Institute for Communicable Diseases, Sandringham, Johannesburg 2131, South Africa; 4 Department of Immunology, The Scripps Research Institute, 10666 North Torrey Pines Rd. La Jolla, CA 92037, USA; 5 Department of Immunology and Molecular Biology, The Scripps Research Institute, 10666 North Torrey Pines Rd. La Jolla, CA 92037, USA; 6 Department of Molecular Biology and The Skaggs Institute for Chemical Biology, The Scripps Research Institute, 10666 North Torrey Pines Rd. La Jolla, CA 92037, USA

## Abstract

During natural HIV infection, an array of host receptors are thought to influence virus attachment and the kinetics of infection. In this study, to probe the interactions of HIV envelope (Env) with various receptors, we assessed the inhibitory properties of various anti-Env monoclonal antibodies (mAbs) in binding assays. To assist in detecting Env in attachment assays, we generated Fc fusions of full-length wild-type gp120 and several variable loop-deleted gp120s. Through investigation of the inhibition of Env binding to cell lines expressing CD4, CCR5, DC-SIGN, syndecans or combinations thereof, we found that the broadly neutralizing mAb, 2G12, directed to a unique carbohydrate epitope of gp120, inhibited Env-CCR5 binding, partially inhibited Env-DC-SIGN binding, but had no effect on Env-syndecan association. Furthermore, 2G12 inhibited Env attachment to primary monocyte-derived dendritic cells, that expressed CD4 and CCR5 primary HIV receptors, as well as DC-SIGN, and suggested that the dual activities of 2G12 could be valuable *in vivo *for inhibiting initial virus dissemination and propagation.

## Background

The envelope glycoprotein (Env) of HIV mediates virus fusion and entry into susceptible cells [1]. Env consists of a trimer of gp120/gp41 heterodimers, in which gp120 is the external surface subunit (SU) responsible for engaging cellular receptors and gp41 is the transmembrane subunit (TM) that mediates membrane fusion [1]. Infection occurs after sequential interactions of gp120 with cellular CD4 and a coreceptor, usually CCR5 or CXCR4. Because of its role in the infection process, Env is the principle target for neutralizing antibodies (nAbs). Unfortunately, very little progress has been made to date in developing vaccines able to elicit nAbs. The hope that one day these efforts may be fruitful is provided by the finding of a few broadly and potently neutralizing mAbs. These include MAb b12, which binds to an epitope overlapping the CD4 binding site of gp120 [2]; 2G12, which binds a cluster of high mannose residues on the immunologically "silent" face of gp120 [3-7]; and Z13, 2F5 and 4E10, which recognize adjacent epitopes in the membrane proximal external region of gp41 [8-13]. Understanding the activities of these naturally occurring nAbs may yield clues as to how to best present their epitopes in vaccines.

The first step in the HIV life cycle is attachment to target cells. Attachment can be achieved by the primary receptors that the virus uses to gain entry to cells. Indeed, for HIV strains adapted for growth in T cell lines, neutralization appears to be based entirely on inhibition of attachment [14-17]. However, for other cell targets, alternative surface molecules can facilitate virus adsorption and modulate the efficiency of the entry process [14,18-21]. For example, neutralization by a blockade of CD4 binding does not impair virus attachment to peripheral blood mononuclear cells (PBMCs) [22], suggesting the involvement of interactions other than gp120-CD4 in initial virus attachment [15,18,23]. Furthermore, due to low CD4 expression, HIV attachment to macrophages and dendritic cells is completely dependent on supplementary receptors [19].

Three main classes of HIV attachment receptors have been found to modulate HIV entry via CD4 and chemokine receptors: LFA-1 [24], DC-SIGN (dendritic cell-specific intercellular adhesion molecule-3 grabbing nonintegrin) [25] and heparan sulfate proteoglycans (HSPGs) [14]. Though attachment can involve molecules other than Env that are incorporated into the virus membrane [26-30], as exemplified by LFA-1-ICAM-1, from an intervention perspective, interactions involving Env are of greater interest.

DC-SIGN is a mannose-specific, calcium-dependent (C-type) lectin specifically expressed on dendritic cells (DCs) that plays a key role in the development of immune responses to highly glycosylated viral pathogens, including primate lentiviruses [25,31]. DC-SIGN captures virus via through N-linked high mannose structures on gp120, after which the dendritic cell transports the virus to secondary lymphoid tissue. In normal circumstances, this would facilitate a strong antiviral immune response. However, for HIV-1, transport to lymph nodes has the unfortunate side effect of presenting the virus to primary CD4^+ ^T cell targets, facilitating trans-infection and virus dissemination throughout the body [21,25,31-34]. Overall, the very high (low nanomolar) affinity of DC-SIGN for gp120 [35,36] and the presence of DCs in mucosal surfaces suggest a key role for DC-SIGN in virus transfer from the submucosa to secondary lymphoid organs during sexual transmission [37].

HSPGs are transmembrane receptors expressed in high concentrations on the surface of adherent cells (e.g. epithelial cells, endothelial cells and macrophages), but not suspension cells (e.g. T-lymphocytes). HSPGs were first reported to mediate HIV attachment to the adherent cell line, HeLa [17,38,39]. Though fresh macrophages generally express low levels of HSPGs, a single family of HSPGs, the syndecans, present on monocyte-derived macrophages (MDMs) have been shown to mediate HIV binding [19,20]. Syndecans may also contribute to attachment to PBMCs, despite relatively low expression, [18,40]. Although syndecans can bind HIV virions lacking Env, in part through binding to cyclophilin A present on the virus surface [19,41], most virus attachment appears to be gp120-specific, especially for PBMC-produced virus [17,20,42]. Just as DC-SIGN-expressing DCs capture and transport virus to the lymph node and propagate CD4 T cell infection *in trans*, so can syndecan-expressing macrophages. These molecules can also facilitate infection *in cis*. That is, when expressed on cells that also bear CD4 and coreceptor, they markedly enhance virus entry. In this way, DC-SIGN and HSPGs effectively increase the tropism of HIV by concentrating virus where primary receptor levels are otherwise below the threshold required for efficient entry, thereby promoting virus dissemination [20,31].

The nature of the interactions of CD4, CCR5, DC-SIGN and HSPGs with HIV-1 Env during infection have implications for intervention strategies. Blocking Env-based virus attachment to any of these cellular receptors would provide a rationale for new microbicide or vaccine strategy. Understanding how Env interacts with these receptors and, moreover, how presently available monoclonal antibodies (mAbs) inhibit these interactions would be a step toward this goal. Studies to date have revealed that mAb b12 blocks gp120-CD4 binding [2]; mAbs directed to gp120 epitopes that are induced by sCD4, as well as V3 mAbs, interfere with CCR5 binding [43-45]; and 2F5 and 4E10 appear to prevent fusion events that occur after CD4 and CCR5 binding, though they may also bind to Env in its native form [12,46]. In comparison, relatively little is known about how these mAbs might block virus attachment to various cells, and indeed what Env determinants are important (12). This is due in part to difficulties in unequivocally measuring virus-cell binding. For example, measuring mAb inhibition of virus attachment is complicated when the virus is also neutralized by the mAb. Thus, methods to measure attachment have tended to rely on detecting surrogates of virus binding, such as virus-associated p24 or surface HLA-DR [16,19,47,48], or the use of immunofluorescent-labeled virus [14,49]. Measuring attachment is further complicated by the differential expression of attachment molecules on target cells and differential incorporation of adhesion molecules on the virus particle, depending on the producer cells. Thus, when investigating virus-cell attachment, a careful consideration of how expression of surface proteins might differ between various virus producer and target cells is crucial [20].

In light of the difficulties in assaying virus attachment, investigating Env-receptor interactions using monomeric gp120 offers a convenient alternative. Although gp120 monomers may not fully recapitulate the properties of oligomeric Env on virus, gp120 binding studies provide a useful adjunct to whole virus attachment studies by unequivocally measuring Env's contribution in the absence of any confounding background attachment by virus-cell interactions not involving Env. Nevertheless, soluble Env binding assays come with their own technical challenges. For example, Env binding has been detected either by a radioactive readout [50] or by lysis of target cells, gp120 immunoprecipitation and autoradiography [51-53]. Binding assays would, therefore, be simplified by using gp120 that is directly tagged to simplify its detection. Previously, the Fc portion of IgG has been genetically fused to several proteins of clinical interest, including cytokines [54,55] and viral envelope proteins [56-59]. Not only does Fc-fusion provide a very convenient way to express and purify proteins of interest (using protein A or G), but it also provides a convenient way to directly tag the protein of interest, avoiding any difficulties associated with secondary detection methods.

Fc-Env chimeras have been previously used to assist traditional virus-cell binding assays to simplify the study of Env attachment and Env-receptor interactions [36,60]. MAbs directed to the V3 loop and CD4-induced epitopes were found to inhibit virus attachment to syndecans but not to DC-SIGN [20]. We, and others previously used Fc-gp120 chimeras to map the determinants of the V3 loop important for attachment of virus to syndecan-expressing cells [42]. Other ELISA data suggested a role for the V3 loop in DC-SIGN-gp120 binding [61]. However, this finding is difficult to reconcile with the binding of V3 loop-deleted gp120 to DC-SIGN [42,61].

Therefore, we used Fc-gp120 fusion proteins to probe Env binding to cellular receptors and examine the abilities of various mAbs to inhibit Env binding to various cell lines with well-defined expression profiles of CD4, CCR5, DC-SIGN and HSPGs. We focused on 2G12 because, compared to other nAbs, relatively little is known about its mechanism and also because its unusual carbohydrate epitope does not overlap that of any other known anti-gp120 mAb, suggesting that it may have a unique mode of action [62]. To assess the in vivo relevance of our findings, we also investigated mAb inhibition activities using primary monocyte-derived DCs (MDDCs) and peripheral blood lympocytes (PBLs) as targets.

## Results

### Characterization of Fc-gp120 chimeras

**Figure 1:**
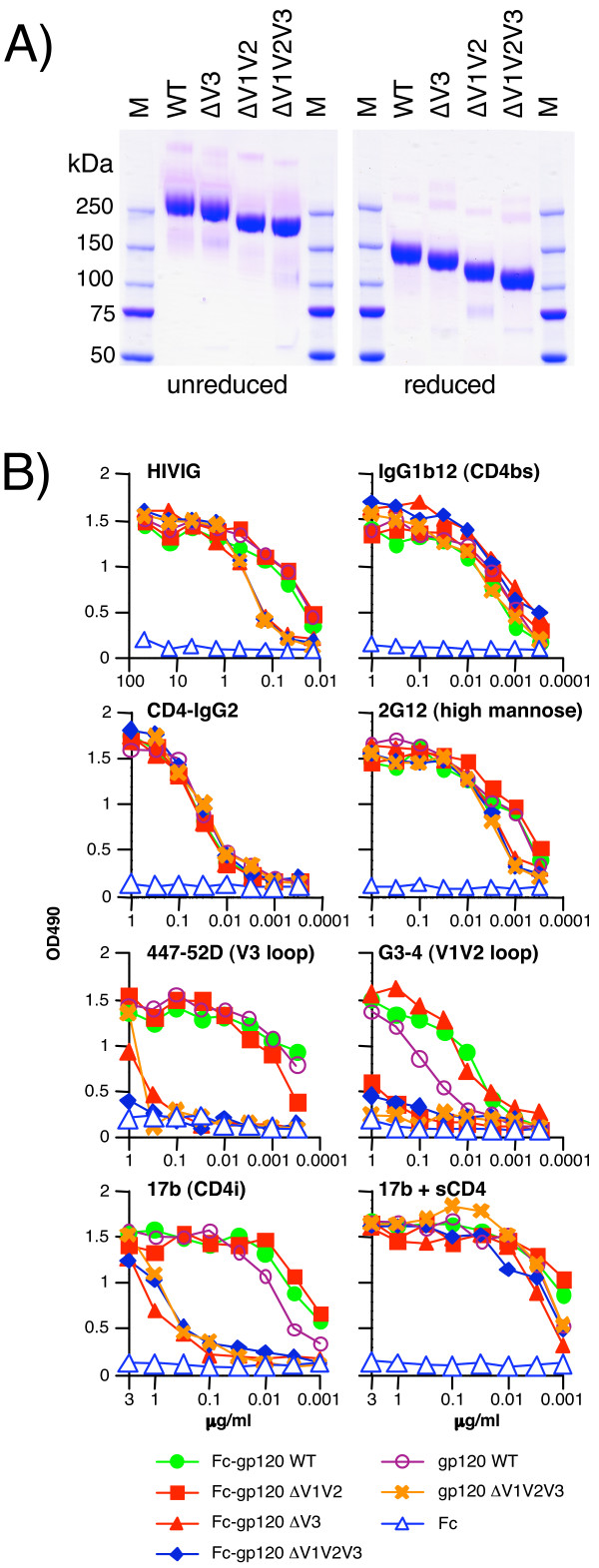
**Design and production of the Fc-gp120 chimeras**. Fc-gp120 chimeras were constructed by fusing the C-terminus of human IgG1 Fc (H, CH2, CH3 domains) in-frame with the N-terminus of gp120_JR-CSF_. Full-length wild-type (WT) and ΔV3, ΔV1V2 and ΔV1V2V3 forms of Fc-gp120 were expressed. A) SDS PAGE and Coomassie staining of purified Fc-gp120 chimeras under reducing and non-reducing conditions. B) ELISA assays comparing mAb binding to Fc-gp120 chimeras, an Fc control and monomeric full-length gp120 WT and ΔV1V2V3. Symbols are as indicated. Results are representative of two independent assays.

Purified Fc-gp120 chimeras were first analyzed by SDS-PAGE in non-reducing (Fig. 1A, left panel) and reducing (Fig. 1A, right panel) conditions. Fc-gp120 WT dimers have a molecular weight of ~290 kDa in non-reducing conditions and ~145 kDa in reducing conditions. The variable loop-deleted versions of Fc-gp120 behaved in a similar manner. Thus Fc-gp120 chimeras exist as homodimers, presumably stabilized by non-covalent association of the Fc portion. To address whether linkage of Fc to gp120 or dimerization affects gp120 conformation, Fc-gp120 chimeras were compared to monomeric gp120WT, ΔV1V2V3 and an Fc fragment, by ELISA (Fig. 1B). MAb b12 and CD4-IgG_2 _bound equivalently to all of the gp120-containing constructs. HIVIG and 2G12 bound somewhat more weakly to constructs lacking the V3 loop. As expected, the V3 loop-binding mAb 447-52D did not recognize any of the ΔV3 gp120 proteins. Also as expected, the V2-specific mAb, G3-4, did not recognize constructs that lacked V1V2 loop domains. The CD4i mAb 17b bound relatively weakly to the V3 loop-deleted gp120s, but sCD4 restored high affinity CD4i mAb binding to all constructs. Overall, the ELISA data revealed similar mAb binding patterns for full-length and variable loop-deleted forms of Fc-gp120 dimers and gp120 monomers. Therefore, Fc-gp120 fusion and dimerization had little effect on gp120's immunochemical properties, validating them as useful tools for studying Env-receptor interactions.

### Fc-gp120 binding to cellular CD4 and its inhibition

**Figure 2:**
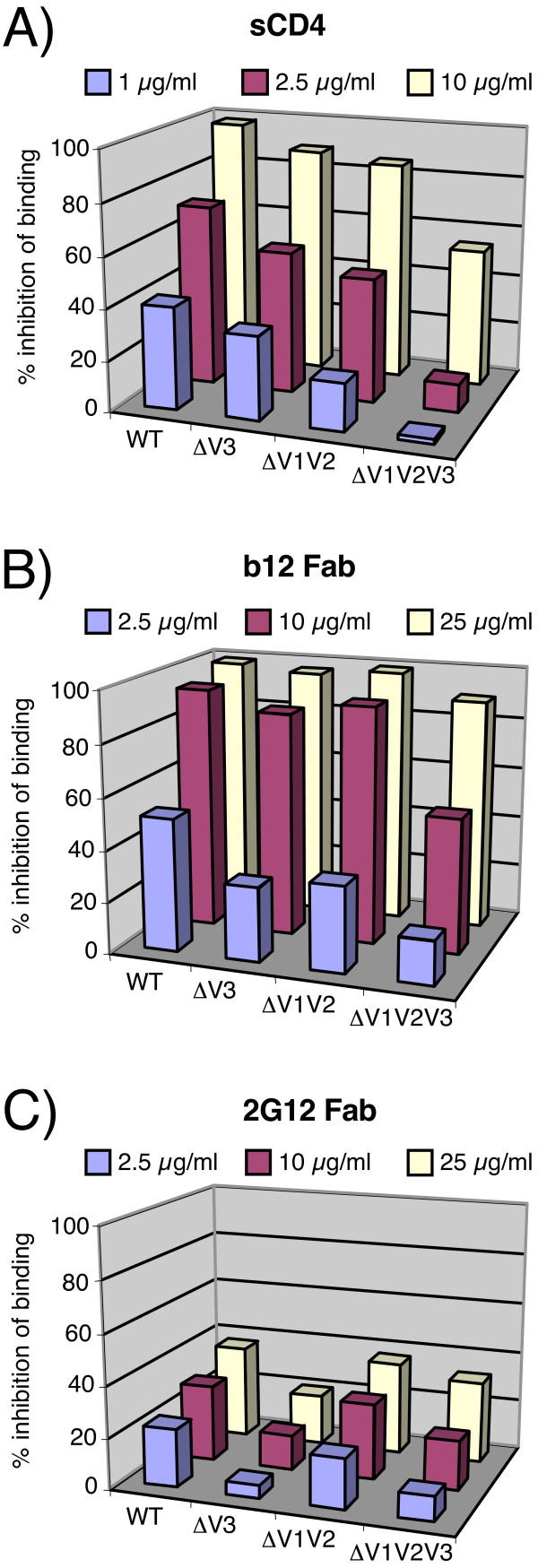
**Binding of Fc-gp120 chimeras to cellular CD4**. Inhibition of binding of Fc-gp120 chimeras to CEM-CD4 cells by (A) sCD4, (B) Fab b12 or (C) Fab 2G12. Inhibition of binding was assessed by FACS analysis and is expressed as % binding inhibition. Results are means of triplicate determinations.

We next analyzed Fc-gp120 binding to receptor-bearing cell lines by FACS, initially using CEM cells expressing CD4 but no coreceptor. We developed a competition format to measure inhibition of Fc-gp120 binding by pre-incubating Fc-gp120 with graded concentrations of soluble ligands (sCD4, b12 and 2G12). We found that sCD4 inhibited Fc-gp120 binding in a dose-dependent manner, with nearly 100% inhibition at 10 μg/ml sCD4 for WT, ΔV3 and ΔV1V2 Fc-gp120 (Fig. 2A). Interestingly, the competition efficiency was diminished for ΔV1V2V3 Fc-gp120, with only 50% inhibition at 10 μg/ml sCD4. This was unexpected, considering that ΔV1V2V3 was recognized equivalently to the other Fc-gp120 chimeras by CD4-IgG_2 _(Fig. 1C), but might stem from differences in affinity of different forms of CD4 (sCD4, CD4-IgG_2 _and cellular CD4) for Fc-gp120 ΔV1V2V3. We next investigated other mAb inhibitors, ensuring that detection via the Fc domain solely reflected Fc-gp120 binding, by using Fab fragments. We found that the b12 Fab, directed to an epitope overlapping the CD4 binding site, inhibited Fc-gp120 binding, as expected (Fig. 2B). In contrast, 2G12 Fab did not efficiently inhibit Fc-gp120 binding to CEM cells (Fig. 2C), suggesting that the mAb inhibits an event after gp120-CD4 attachment. 2G12 was even weaker at inhibiting the binding of V3 loop-deleted Fc-gp120s (ΔV3 and ΔV1V2V3) to CD4^+ ^cells (Fig. 2C), consistent with its above noted inefficient binding to V3 loop-deleted Fc-gp120s [63] (Fig. 1B).

### Fc-gp120 binding to cellular CCR5 and its inhibition

**Figure 3:**
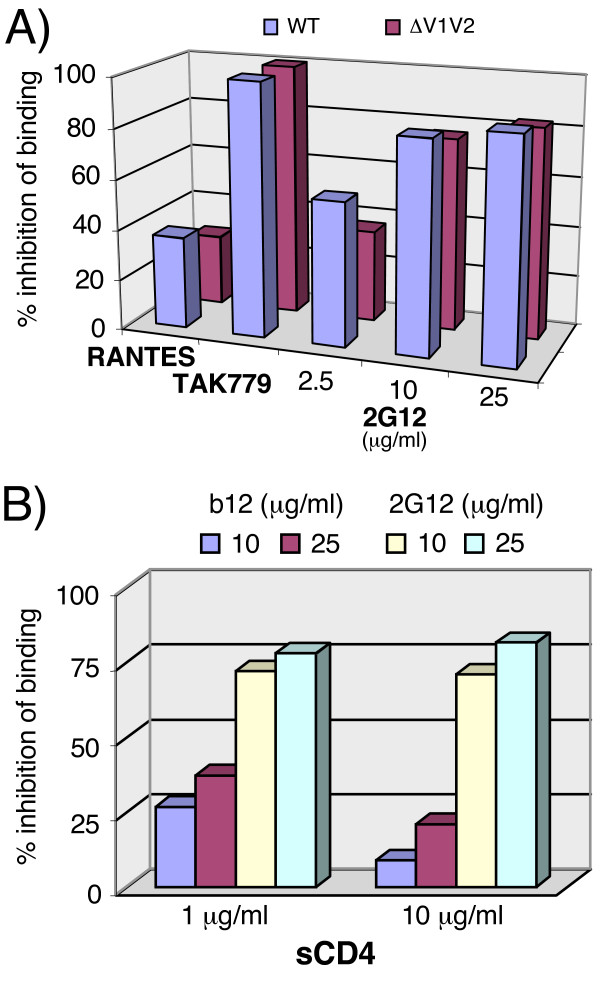
**Binding of Fc-gp120 chimeras to cellular CCR5**. A) Inhibition of Fc-gp120-sCD4 complex binding to CCR5 by RANTES (1 μg/ml), TAK779 (0.5 μg/ml) and 2G12. Binding inhibition was assessed by FACS analysis and is expressed as a percentage calculated with reference to m.f.i. data using Fc-gp120-sCD4 complexes alone (0% inhibition value) and background with nothing added (100% inhibition value). Results are the means of triplicates. B) Inhibition assays were performed using Fc-gp120 complexed with 1 or 10 μg/ml of sCD4 and mAbs 2G12 or b12 at 10 or 25 μg/ml. Inhibition of binding was expressed as a percentage, as above. Results are the means of duplicates.

Further investigation of the Fc-gp120/sCD4 complex binding to CCR5^+ ^cells, as expected, showed specific inhibition by CCR5 binding molecules RANTES and TAK779 (Fig. 3A). Based on previous studies, inhibition of Fc-gp120 binding to CCR5 might be expected by mAb directed to CD4-induced epitopes [50,51] or the V3 loop [44,64]. However, mAb 2G12 directed to a cluster of high mannose oligosaccharides in the "silent domain" of gp120 [4,5,7], is distinct from the domains so far directly implicated in either CD4 and CCR5 binding [65,66] and, therefore, neutralization is thought to occur by steric inhibition [6,50]. Although 2G12 does not significantly interfere with CD4-gp120 binding (Fig. 2), there have been hints of a possible CCR5 blocking mechanism [50,67-69]. To further investigate, we examined 2G12's effect on Fc-gp120-sCD4 complex binding to CCR5. We found that 2G12 inhibited this interaction (Fig. 3A), approaching 100% effectiveness at 25 μg/ml, consistent with the previously reported 2G12 IC90 neutralizing titer of ~5 μg/ml against JR-CSF [60].

Although our previous experiments indicated that 2G12 does not interfere with Fc-gp120 binding to *cellular *CD4 (Fig. 2C), to rule out any direct competition between 2G12 and sCD4 in the CCR5 binding assay, we performed additional inhibition assays using 1 μg/ml or 10 μg/ml of sCD4 to form complexes with Fc-gp120 WT, with mAb b12 as a control. As expected, higher concentrations of sCD4 decreased the ability of b12 to inhibit binding of Fc-gp120 to CCR5^+ ^cells (Fig. 3B). In contrast, higher sCD4 concentrations did not diminish 2G12 blocking of the Fc-gp120-sCD4 complex to CCR5^+ ^cells, consistent with the notion that 2G12 inhibits only the gp120-CCR5 interaction (Fig. 3B).

**Figure 4:**
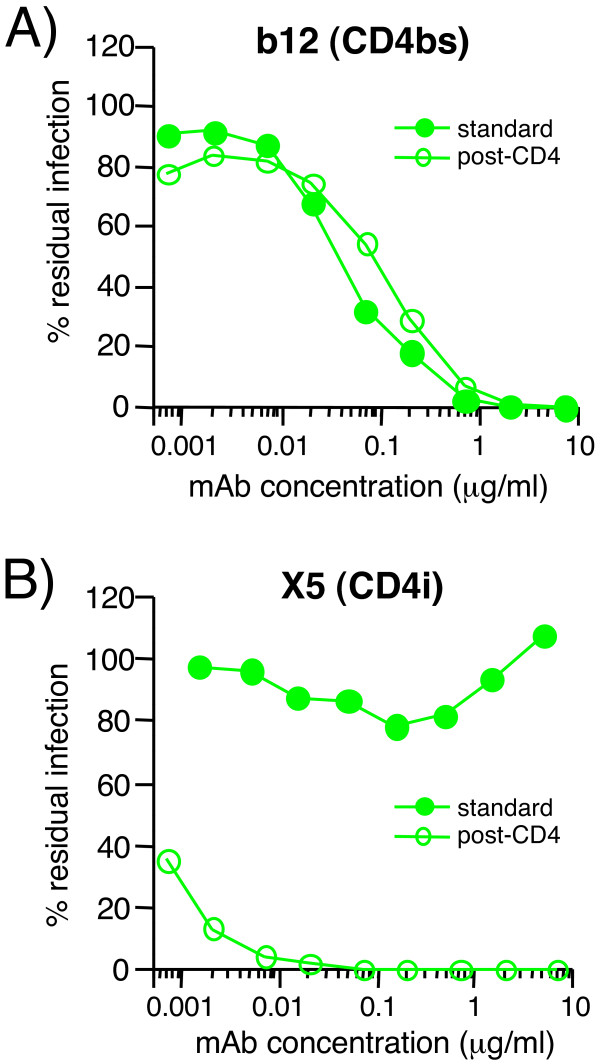
**2G12 neutralizes HIV-1 JR-CSF effectively in a post-CD4 assay format**. The neutralization activity of mAbs A) b12, B) X5, and C) 2G12 was assessed in the standard (closed circles) and post-CD4 (open circles) neutralization formats. Results are expressed as % of residual infection, with 100% representing infection in the absence of mAb. Results are representative of two experiments.

To investigate whether our findings with Fc-gp120 are relevant to virus bearing functional trimers, we examined virus neutralization in standard and "post-CD4" formats (Fig. 4). The standard neutralization format uses Cf2.Th.CD4.CCR5 cells. The "post-CD4" format involves pre-incubating virus with sCD4 and graded concentrations of a nAb, then measuring residual infection of CD4 negative Cf2.Th.synCCR5 cells. Use of target cells expressing only CCR5 eliminated any potential competition between cellular CD4 and sCD4. As expected, the positive control mAb b12 neutralized effectively in the standard format. However, it was approximately 2.5-fold less active in the "post-CD4" format (Fig. 4A), consistent with b12 binding an epitope that overlaps the CD4 binding site on gp120. The sCD4 protein (used at 10 μg/ml) apparently does not completely block b12 neutralization, presumably because at higher b12 concentrations, sCD4 might be displaced. In contrast, mAb X5 did not neutralize effectively in the standard format, but showed extremely potent activity in the "post-CD4" format (Fig. 4B). This is consistent with the notion that the X5 epitope, overlapping the CCR5 binding site of gp120, is cryptic in the native form of gp120, but becomes exposed and induced upon CD4 binding. A third pattern was observed with mAb 2G12, which neutralized the virus in *both *formats, with an approximately 4-fold greater activity in the post-CD4 format. The difference was not as dramatic as observed with X5, implying that the 2G12 epitope is not induced by CD4 binding, but rather that it is impartial. MAb 2G12, therefore, appears to neutralize HIV-1 by steric interference of gp120-CCR5 binding, consistent with CCR5 blocking assays using Fc-gp120 (Fig. 3), validating Fc-gp120 chimeras as adjuncts for further investigating Env-receptor interactions.

### Fc-gp120 binding to DC-SIGN and HSPGs and its inhibition

**Figure 5:**
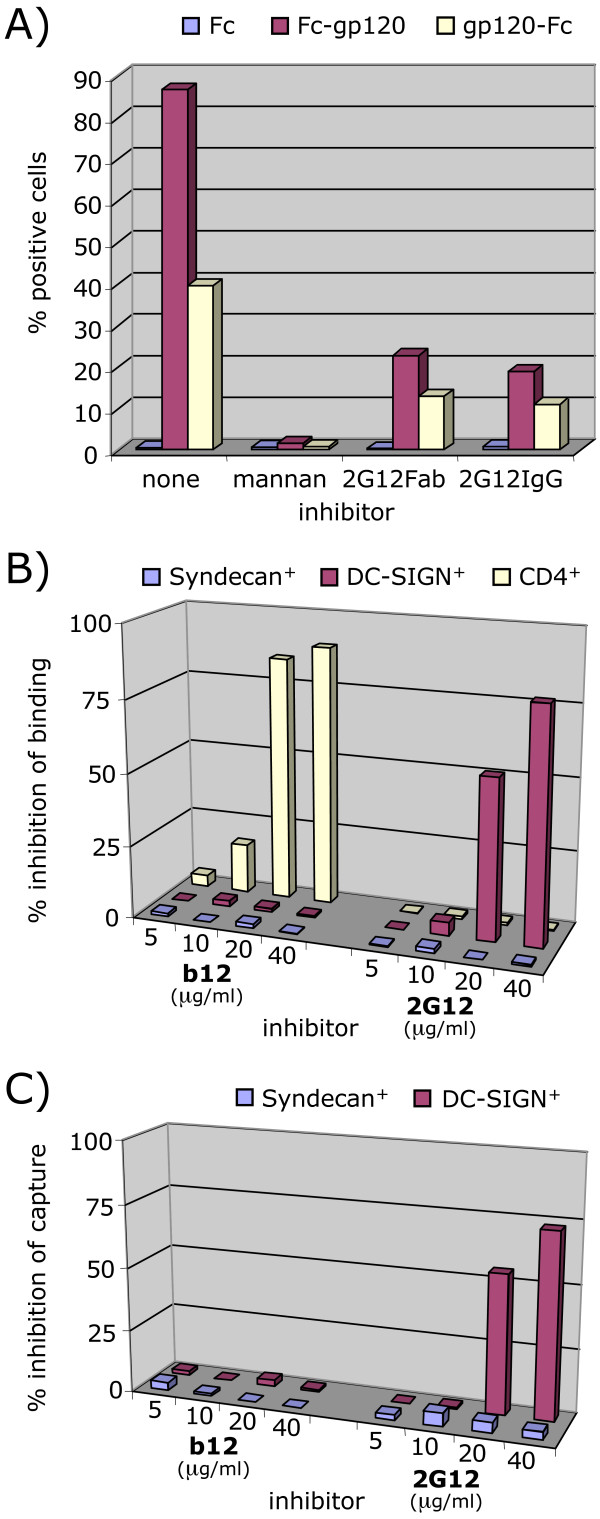
**Effect of 2G12 on Env-HSPG and Env-DC-SIGN binding**. A) The inhibitory effect of mannan (50 μg/ml) and 2G12 Fab (25 μg/ml) and 2G12 IgG (25 μg/ml) on binding of Fc-gp120 and gp120-Fc chimeras to CHO pgsA745 cells transduced with MIGR1 GFP/DC-SIGN. Fc alone was included as a negative control. Data are representative of triplicates. B) Namalwa cells expressing either CD4, HSPGs (syndecan 2) or DC-SIGN were assessed for Fc-gp120 binding in the absence or presence of varying concentrations of mAbs b12 and 2G12. Inhibition is expressed as a percentage of Fc-gp120 binding compared to controls. C) Virus capture by Namalwa cells expressing either HSPGs or DC-SIGN was assessed in the absence or presence of varying concentrations of mAbs b12 and 2G12. Inhibition is expressed as percentage of p24 captured compared to controls. Results are representative of two experiments.

DC-SIGN has been reported to recognize high mannose residues on gp120 [4,5,7]. In a serie of experiments, we initially investigated DC-SIGN-gp120 interaction using Fc-gp120 chimeras and DC-SIGN-expressing CHO pgsA745 target cells deficient in glycosaminoglycan biosynthesis and, therefore, HSPG expression. Elimination of HSPG expression was important, considering that high affinity gp120-HSPG binding might confound any true measurement of gp120-DC-SIGN binding. We found that all of the Fc-gp120 chimeras bound these cells (Fig. 5A, data shown only for Fc-gp120 WT). Fc-gp120 binding to DC-SIGN was specific, since Fc alone did not bind, and Fc-gp120 binding could be blocked by mannan (Fig. 5A) and EGTA (data not shown). Since 2G12 also recognizes terminal mannose residues [60], we wondered if it might also inhibit Fc-gp120-DC-SIGN binding. We found that 25 μg/ml 2G12 IgG inhibited DC-SIGN-Fc-gp120 WT to ~75% (Fig. 5A). The specificity of this inhibition was further supported by the observation that 2G12 Fab and IgG showed similar degrees of competition. Thus, in addition to gp120-coreceptor blocking, 2G12 appears to also inhibit gp120-DC-SIGN interaction. Similar results were observed with the variable loop-deleted Fc-gp120 chimeras (data not shown).

Our results contrast with a previous report that 2G12 had no effect on gp120-Fc binding to DC-SIGN-expressing BC7 cells or on virus binding to DC-SIGN-expressing 293T cells [60]. Several differences between ours and the previous study might explain this discrepancy: i) we used a gp120 chimera in which Fc is positioned at the N-terminus of gp120 (Fc-gp120), whereas Hong *et al. *used a chimera with the Fc domain fused at the C-terminus of gp120 (gp120-Fc); ii) the previous analysis was performed using 293T cells that, characteristic of adherent cell lines, express relatively high levels of HSPGs that can also bind gp120 and might, therefore, have masked the inhibitory effect of 2G12 on gp120 binding to DC-SIGN; iii) producer cell glycosylation machinery may influence gp120 glycan structure and neutralization sensitivity. Our Fc-gp120 chimeras were produced in CHO cells, whereas previously, wild-type gp120-Fc was produced in 293T cells [60]. Furthermore, gp120 fusion with Fc at the N-terminus rather than the C-terminus leads to more uniform glycosylation; iv) our gp120 chimeras were protein A purified, whereas Hong *et al. *used unpurified gp120-Fc supernatants.

To investigate point i), whether the position of Fc influences 2G12 inhibition, we assessed the ability of 2G12 to inhibit gp120-Fc-DC-SIGN interaction. Although preliminary experiments indicated that gp120-Fc bound to CEM.CD4^+ ^cells with similar affinity as Fc-gp120 (data not shown), Fc-gp120 bound more efficiently to DC-SIGN than did gp120-Fc (Fig. 5A). Nevertheless, 2G12 inhibited gp120-Fc binding by an average of 70%, similar to the inhibition observed with Fc-gp120 (Fig. 5A).

To investigate point ii), whether the discrepancy could relate to HSPG-gp120 binding, we analyzed Fc-gp120 binding to Namalwa cells expressing either CD4, DC-SIGN, or HSPG (Syndecan) (Fig. 5B). Like the CHO pgsA745 cells, Namalwa cells were selected because of the absence of HSPG expression on their surfaces. 2G12 had very little effect on Fc-gp120 binding to CD4^+ ^Namalwa cells, although mAb b12 was able to inhibit, consistent with our earlier findings (Fig. 2C). On Namalwa cells expressing DC-SIGN, as with the CHO pgsA745 DC-SIGN^+ ^cells, 2G12 inhibited Fc-gp120 binding in a dose-dependent manner (Fig 5B). However, 2G12 did not inhibit Fc-gp120 binding to cells expressing HSPG. The results are consistent with the role of the V3 loop rather than the 2G12 epitope in HSPG binding [60], as verified by a lack of binding of V3 loop-deleted Fc-gp120 to HSPG-expressing Namalwa cells (data not shown). We further investigated 2G12 inhibition of whole virus binding to DC-SIGN and HSPGs (Fig. 5C). Consistent with the above results using Fc-gp120, we observed a dose-dependent inhibition of HIV-1 attachment to Namalwa cells expressing DC-SIGN, but no inhibition using cells expressing HSPG (Fig. 5C). We conclude that the lack of 2G12 effect on Env binding to DC-SIGN previously observed [20] may have stemmed from the masking effect of baseline HSPG expression on the target cells. Another study [61] reported that 2G12 did not inhibit virus attachment to DC-SIGN-expressing cells. However, in that study, 2G12 was used at 10 μg/ml, a concentration that is just below the threshold required to inhibit gp120-DC-SIGN binding (Fig. 5C). Overall, we have shown that 2G12 inhibits the interaction of virus with DC-SIGN expressed on various cell types and that the virus-DC-SIGN association can be modeled effectively, if imperfectly, by measuring Fc-gp120-DC-SIGN binding.

### ELISA mapping of the determinants of gp120 involved in DC-SIGN binding

**Figure 6:**
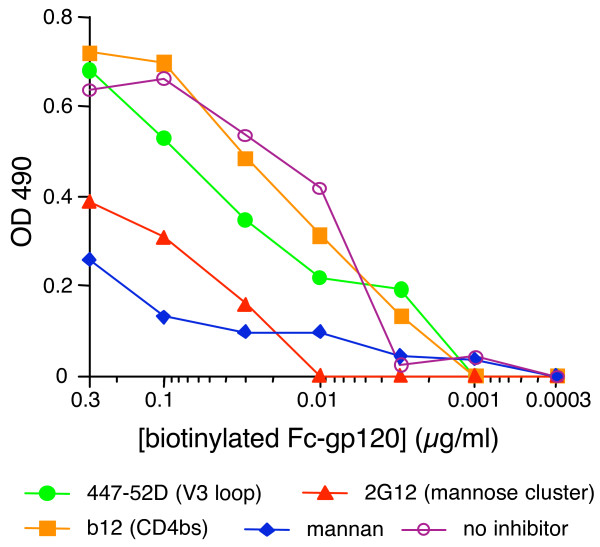
**2G12 inhibits DC-SIGN-gp120 interaction in ELISA**. The ability of mAbs to inhibit DC-SIGN-gp120 interaction was evaluated. Fc-DC-SIGN was coated on the plate. Fixed concentrations of mAbs or mannan were then added, followed by graded concentrations of biotinylated Fc-gp120, which was detected using a streptavidin-alkaline phosphatase conjugate. Results are representative of two independent assays.

Previously published ELISA MAb competition studies concerning the determinants of gp120 important for DC-SIGN interaction and unexpectedly found that V3 loop mAbs interfered with gp120-DC-SIGN binding, but 2G12 did not [61]. However, a direct role of the V3 loop in DC-SIGN binding is unlikely, because ΔV3 and ΔV1V2V3 Envs bind effectively to DC-SIGN-expressing cells, as probed using CHO pgs-A745 cells (data not shown), Namalwa cells (Fig 5), MDDCs (see below), and others [61]. Since CHO pgsA745 and Namalwa cells are deficient in CD4, CCR5 and HSPG receptors that might foster the binding of V3-deleted Envs, the inhibition of gp120-DC-SIGN binding by mAbs is a paradox worth reinvestigating. We, thus, devised two competitive ELISA formats. In a format similar to that reported previously [60], the V3 loop mAb 447-52D inhibited Fc-DC-SIGN binding to gp120 (not shown), consistent with the previous report. In contrast, 2G12 was relatively ineffective and b12 had no effect. Even mannan, used as a positive control, was only moderately effective, perhaps suggesting a problem with this format. Considering our FACS data implying that 2G12 inhibits DC-SIGN binding to gp120 (Fig. 5), these ELISA results were unexpected. Therefore, we further investigated the DC-SIGN-gp120 interaction in essentially in the reverse ELISA format. Here, Fc-DC-SIGN was coated on the ELISA wells, fixed concentrations of mAb competitors were then added and biotinylated Fc-gp120 that had been previously titrated on a separate plate was overlaid, the binding of which was then detected using a strepatavidin-alkaline phosphatase substrate. This format gave remarkably different results (Fig. 6). The IC50 of Fc-gp120 binding was ~5 ng/ml (~0.017 nM, based on a molecular weight of ~290 kDa for Fc-gp120), an order of magnitude higher than the IC50 the other ELISA format. In addition, the competitive strength of mAbs and mannan were also greater and background binding was lower. Moreover, the competition profile was qualitatively different. Mannan convincingly inhibited the gp120 binding to DC-SIGN, as would be expected. In contrast to the other format, 2G12 was highly effective, and a V3 loop mAb 447-52D was largely ineffective, as was b12 (Fig. 6). The basis for the difference between these ELISA formats is unclear. It is possible that the competition in the new format was stronger due to the use of whole IgGs instead of Fabs. However, this does not explain the qualitative differences, especially considering that, in the new format, Fab 2G12 competition was even greater than the whole IgG (not shown). Instead, it is likely that coating gp120 to the ELISA plate interfered with DC-SIGN binding in the initial ELISA format. Considering the higher affinity gp120-DC-SIGN binding and that the competition analysis was consistent with our results in Fig. 5, we suggest that the ELISA format shown reflects the true gp120-DC-SIGN binding relationship – partially inhibitable by 2G12, but not by V3 loop-specific mAbs.

### The relevance of mAb inhibition using primary cells

**Figure 7:**
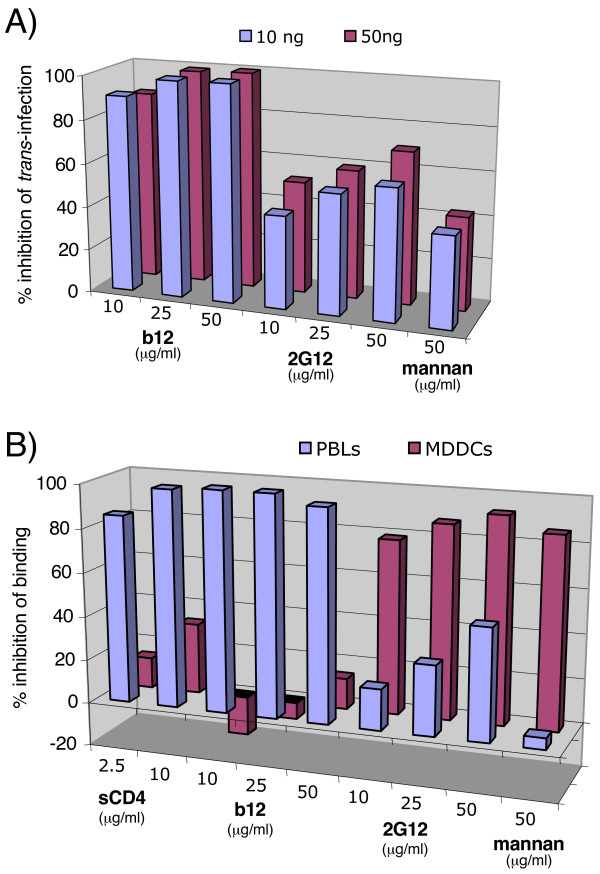
**Inhibition of trans-infection by MDDCs and of Fc-gp120 binding to primary PBLs and MDDCs**. A) MAbs b12 or 2G12, or mannan were incubated with virus (10 and 50 ng p24). The mixture was then incubated with primary MDCCs, followed by washing. Cells were then added on top of TZM-BL cells previously plated a day before, and trans-infection was assayed two days later by a β-gal assay. Results are the mean values of triplicates. B) Inhibition of Fc-gp120 binding to primary PBLs and MDDCs in the presence of sCD4, b12 Fab, 2G12 Fab or mannan at the concentrations indicated. Inhibition is expressed as the percentage of Fc-gp120 binding.

We next assessed the *in vivo *relevance of the modes of 2G12 inhibition using primary monocyte-derived dendritic cells (MDDCs) generated in-vitro. Studies of MDDCs have emphasized the importance of mannose C-type lectin receptors (MCLRs), particularly DC-SIGN, for gp120 binding, and, therefore, virus uptake and dissemination to CD4^+ ^T cells. To investigate whether 2G12 could inhibit DCs transfer to CD4^+ ^T cells, we performed trans-infection experiments using primary immature MDDCs and TZM-BL cells. TZM-BL cells are HeLa cells engineered to express CD4 and CCR5, and to harbor a β-galactosidase gene under the control of the HIV LTR (see materials and methods section). As shown on Figure 7A, MAbs b12 and 2G12 effectively inhibited trans-infection of virus from primary MDDCs. Mannan also inhibited trans-infection by preventing virus capture on MDDCs. Thus, while b12 inhibition occurred by classic neutralization of captured virus, the activity of 2G12 could stem from both the inhibition of virus attachment via DC-SIGN, or another C-type lectin expressed on MDDCs, as well as by blocking virus binding to CCR5. To further investigate, we examined mAb inhibition of Fc-gp120 binding to MDDCs and PBLs. Our results revealed that mannan, EGTA (not shown), and 2G12 partially inhibited Fc-gp120 attachment to MDDCs, the latter with an IC50 of ~20 μg/ml (Fig. 7B), but b12 and sCD4 did not significantly inhibit binding. In contrast, on PBLs, b12 and sCD4 were effective at inhibiting binding, and 2G12 was only partially effective. Taken together, our results suggest that Env attachment via DC-SIGN or other MCLRs expressed on MDDCs supersedes attachment by primary receptors. In contrast, for primary PBLs, attachment appears to occur predominantly via CD4 and CCR5.

## Discussion

In the present study, we investigated mAb interference of Env and virus attachment to various cell lines and primary lymphocyte targets. Fc-gp120 was shown to be a relevant tool to investigate gp120-receptor interactions without complications of neutralization and other difficulties in detection associated with whole virus binding assays. Fc-gp120 has several advantages over the use of live virus, in providing a rapid (FACS and ELISA-compatible), convenient (easy to purify and detect) and safe (non-infectious) tool for assessing of gp120-receptor interactions. ELISA data (Fig. 1) indicated that the structural integrity of gp120 in the Fc-gp120 chimeras was conserved and that the Fc domain had no deleterious effects on gp120 folding. Furthermore, fusing Fc upstream of gp120 was useful, since it allowed greater expression, compared to downstream fusion.

Using Fc-Env chimeras as a molecular tool, we investigated gp120's interaction with its receptor, coreceptor and attachment cofactor(s), and the inhibition mechanism of nAbs and entry inhibitors. We observed inhibition of WT and loop-deleted Fc-gp120 chimera binding to CD4 by sCD4, b12 and to CCR5-expressing cells by 2G12 (Figs. 2 and 3). These results were consistent with the mAbs activities against whole virus in neutralization assays (Fig. 4). Similarly, 2G12 inhibition of Fc-gp120-DC-SIGN paralleled its inhibition of virus binding to DC-SIGN (Fig. 5). Although Fc-gp120 is a useful tool, we acknowledge that inhibition of its binding to cells might not always predict a biologically relevant activity against intact virus, because conformational constraints restrict many non-neutralizing Abs from binding to trimers but not gp120 monomers. For example, a non-neutralizing mAb directed to the gp120 CD4 binding site might inhibit Fc-gp120 binding to CD4^+ ^cells, but would not affect the interaction of virus with CD4. Thus, inhibition of Fc-gp120 binding to receptors may be necessary, but not be sufficient to predict inhibition of whole virus attachment. On the other hand, it has been recently reported that virus particles themselves may bear gp120/gp41 monomers [70], perhaps increasing the relevance of Fc-gp120 as a surrogate for viral Env. This possibility is supported by the similar inhibition IC50s of Fc-gp120 and virus binding to DC-SIGN expressing cells by 2G12 (Figs 5B and 5C). Indeed, Fc-gp120 chimeras might provide tools for rapid and convenient screening of small molecule inhibitors and nAbs able to inhibit gp120-receptor interactions that could be useful in HIV therapies or microbicides. Indeed, for effective use in ELISA, the Fc portion, with its numerous positively charged residues, binds preferentially to microwells, leaving gp120 free to interact with a ligand.

Our most significant findings were that 2G12 inhibits HIV-1 by two mechanisms: blocking both gp120-CCR5 and gp120-DC-SIGN interactions. 2G12 inhibition of gp120 binding to DC-SIGN was revealed in several distinct assays: inhibition of both Fc-gp120 and whole virus binding to two different DC-SIGN-expressing cell lines and inhibition of binding to recombinant DC-SIGN binding in ELISA. Previously, gp120-DC-SIGN inhibition was not observed [50,51,66]. The discrepancy probably relates to baseline expression of HSPGs on target cells that can also mediate Env binding. Regarding potency, 2G12 inhibited Env-DC-SIGN interaction with an IC50 of ~20 μg/ml, which is somewhat weaker than its neutralizing IC50 (~5 μg/ml). This difference might be related to the fact that the binding sites of 2G12, DC-SIGN, and CCR5 on Env are each unique. The CCR5 binding site of gp120 most closely approximates the epitope of CD4-induced mAbs on gp120 [4,7,60,71], 2G12 recognizes terminal mannose of a specific array of carbohydrates on gp120, and DC-SIGN binds to gp120 carbohydrates in a manner that is largely independent of specific carbohydrate arrays [60]. Indeed, mutational analysis revealed that removal of carbohydrates that eliminate 2G12 binding do not affect the binding of DC-SIGN [72]. Thus, it is reasonable that 2G12 binding is only partially able to block multimeric DC-SIGN binding [18,20,25]. This adds to the complex and sometimes unexpected competitive relationships of sugar-binding gp120 ligands. So far, information suggests that cyanovirin inhibits 2G12 binding to Env [3,60], but 2G12 does not inhibit cyanovirin binding [3,7]; DC-SIGN-Env binding does not inhibit 2G12 binding [60], but according to present data, the reverse competition appears to be at least partially true.

Overall, our results confirm that initial attachment of HIV to primary cell types may or may not be CD4 dependent [20], depending on the cell type involved. DC-SIGN mediates a dominant role in virus attachment to MDDCs, just as syndecans expressed on MDMs, as well as other mannose C-type lectin receptors (MCLRs) expressed on different DC subsets [73], most importantly the mannose receptor on dermal DCs and langerin on epidermal langerhans cells, the latters being the primary cells to capture virus during mucosal infection [74]. Thus, DC-SIGN, other MCLRs, and HSPGs may play parallel roles in seeding virus infection, although their relative importance in virus capture and transport to lymph nodes remains to be fully understood [75]. Inhibiting virus-DC-SIGN/MCLRs interaction by blocking certain determinants on gp120 may be a valid intervention strategy. Already, cyanovirin, another inhibitor of gp120-MCLRs attachment, is being considered as a microbicide [76]. The effects of 2G12 reported herein highlight its potential to inhibit virus dissemination during primary infection and support further investigation of its possible use as a microbicide, or in post-exposure prophylaxis [77]. Indeed, it was recently reported that compared to other nAbs, 2G12 passive therapy was relatively potent in limiting HIV resurgence in human volunteers that ceased highly active antiretroviral therapy [78]. It is possible that this increased potency relates to the particular properties of 2G12 in inhibiting trans-infection via gp120-DC-SIGN/MCLRs disruption, as well as in neutralizing via gp120-CCR5 disruption, that together might amplify its activity *in vivo*. However, it should be noted that 2G12 does not recognize most clade C and clade E viruses (5), and, therefore, limits its use as an effective prophylactic agent in settings where these viral subtypes predominate. Further studies should help reveal the full potential of 2G12's dual mechanism of action in inhibiting binding of gp120 to CCR5 and DC-SIGN, and, hence, virus dissemination.

## Methods

### Cloning, protein expression and purification

#### i) Env-based proteins

Fc-gp120 chimeras were constructed by fusing an IgG1 Fc domain N-terminal to gp120_JR-CSF _[42]. A series of Fc-gp120 fusion proteins included one with full-length (wild type; WT) gp120 and 3 that were modified to remove variable loop domains: ΔV1V2 (Δ129–194, according to the amino acid numbering of the LAI isolate, replaced by a GSG linker), ΔV3 (Δ298–329, replaced by a GSGG linker) or ΔV1V2V3 (Δ129–194 + Δ298–329). For WT and V1V2-deleted Fc-gp120s, Fc was fused in-frame to the Leu residue at position 51, whereas, in V3 and V1V2V3-deleted Fc-gp120s, Fc was fused in-frame at the Val residue at position 74. Therefore, the first two gp120 chimeras contain an integral C1 region, confirmed by ELISA using C1 specific antibodies (data not shown), whereas the two latter chimeras do not, as they failed to recognize the C1 antibodies. That difference, however, didn't alter the overall conformation of gp120 as an ELISA assay using CD4-Ig2 showed a similar reactivity profile for all the Fc-gp120 chimeras (Figure 1B). Furthermore, a similar binding curve was observed for all the chimeras by a FACS titration assay using CD4^+ ^CEM cells and primary CD4^+ ^T cells (data not shown). The Fc-gp120 chimeras were produced in CHO cells using the glutamate synthetase expression system as previously described (20). Fc-gp120 was batch purified from culture supernatants on protein A. We also generated chimeras where the position of gp120 and Fc was reversed (gp120-Fc). We also generated full-length and ΔV1V2V3 (Δ128–194 + Δ298–329) JR-CSF gp120 without Fc tags in Drosophila SC2 cells using the vector pRMAH3, using methods described previously [79].

#### ii) Fc-DC-SIGN

The entire ectodomain of DC-SIGN was fused in-frame with the Fc region of IgG1. Fc-DC-SIGN was produced using CHO cells and the glutamate synthetase amplification system as described previously [56].

### Monoclonal antibodies, sera, soluble CD4 and small molecule entry inhibitors

The mAbs employed in these studies included b12 (directed to an epitope overlapping the CD4 binding site (CD4bs) of gp120) [2]; 2G12 (directed to a specific high mannose carbohydrate cluster on gp120) [3-7]; 447-52D (directed to the gp120 V3 loop) [80]; G3-4 (directed to the gp120 V1V2 loop) [81]; 17b and X5 (directed to epitopes that are induced on gp120 by CD4 binding; CD4i) [43]. In some cases, monovalent Fab fragments were produced by papain digestion, according to manufacturer's instructions (Pierce). CD4-based proteins were obtained from Progenics Pharmaceuticals: CD4-IgG_2 _(also known as PRO542; a fusion protein in which the variable domains of IgG are replaced by D1D2 of CD4) [82-84] and 4-domain recombinant soluble CD4 (sCD4). HIVIG, a polyclonal IgG purified from pooled HIV+ donor sera, was a gift from Dr. John Mascola. The carbohydrate derivatives EGTA and mannan were obtained from Sigma. Coreceptor binding inhibitors included RANTES (R&D systems, Minneapolis) and TAK779 (courtesy of the NIH AIDS Research and Reference Reagent Program).

### ELISA

To assay the binding of a panel of mAbs and a polyclonal serum, Fc-gp120 chimeras or monomeric gp120 were directly coated on ELISA plates at 5 μg/ml and subsequent steps were performed as described previously [85], except that binding to Fc-gp120 was detected via an anti-Fab instead of anti-Fc alkaline phosphatase conjugate, to avoid cross-reactivity of the conjugate with the Fc portion of Fc-gp120.

### Competition ELISA to investigate gp120-DC-SIGN binding

Two ELISA formats to investigate DC-SIGN-gp120 interaction were developed: i) Monomeric JR-CSF gp120 was coated on Immulon II plates at 5 μg/ml. Fifty microliters of Fab 447-52D, Fab b12, Fab 2G12 or mannan competitors were then incubated for 30 minutes. Fabs were used at 50 μg/ml and mannan at 100 μg/ml. Fifty microliters of graded concentratons of Fc-DC-SIGN that had been previously titrated on a separate plate was then overlaid. Following washing, bound Fc-DC-SIGN was detected using an anti-Fc alkaline phosphatase conjugate (Accurate) and the AMPAK substrate system. ii) Fc-DC-SIGN was coated on ELISA wells at 5 μg/ml. Fifty microliters of saturating concentrations of 2G12, b12, 447-52D (50 μg/ml) or mannan (100 μg/ml) competitors were then added. Biotinylated Fc-gp120 that had been titrated on a separate plate was then overlaid. Bound gp120 was then detected using streptavidin-alkaline phosphatase conjugate (Vector Laboratories, Burlingame, CA). All binding buffers were supplemented with 2 mM CaCl_2_.

### Cells

CEM cells were obtained from Douglas Richman (University of California, San Diego). CF2ThCD4synCCR5 and CF2ThsynCCR5 are canine cell lines that express high levels of CCR5, through a codon-optimized CCR5 gene [86]. CHO-pgsA745 cells and 293T cells co-expressing GFP and DC-SIGN were previously described [36]. PBMC were isolated from donors using Ficoll gradients. MDDCs were produced from PBMC by MACS separation, using anti-CD14 coated magnetic microbeads, and cultured for 6 days in RPMI supplemented with 10% FBS, 50 ng/ml of IL-4 (Peprotech) and 50 ng/ml of GM-CSF (Peprotech). The peripheral blood lymphocytes (PBLs) remaining from MACS separation were cultured in parallel in RPMI supplemented with 10% FBS, 5 μg/ml Concanavalin A (Sigma) and 100 units/ml of IL-2 (NIH AIDS Research and Reference Reagent Program) and both cell fractions from donors were used in binding assays. Namalwa cells, a human B cell parental line, engineered to express either CD4, HSPG (syndecan-2) or DC-SIGN were previously described [20,42]. TZM-BL cells (also known as JC53-BL cells) were used as target cells for pseudovirus infection assays. TZM-BL is a HeLa cell clone that expresses CD4, CXCR4 and CCR5 and contains Tat-responsive reporter genes for firefly luciferase and β-galactosidase under the control of a HIV long terminal repeat [87].

### Analysis of Fc-gp120 attachment to target cells by FACS

To analyze binding of Fc-gp120 chimeras to target cells, Fc-gp120 was incubated at 1 μg/ml with 2 × 10^5 ^cells for one hr at room temperature (RT) in 100 μl of Earl's Balanced Salt Solution (EBSS, Sigma, St. Louis, MO) containing 0.1% bovine serum albumin (BSA, Sigma), washed once in EBSS and resuspended in 100 μl of EBSS/0.1% BSA supplemented with a phycoerythrin conjugated anti-human Fc antibody (ICN-Cappel, Aurora, OH) at a 1:250 dilution. Samples were then washed and processed on a FACScan (Beckton Dickinson, San Jose, CA). Data were acquired and analyzed with Cellquest (Beckton Dickinson) and FlowJo (Tree Star, San Carlos, CA) software, respectively. For gp120/DC-SIGN interaction, CaCl_2 _was added at a final concentration of 2 mM in the incubation and wash buffers. For gp120/CCR5 interaction, Fc-gp120 was pre-incubated with sCD4 for 30 min at RT. For inhibition studies, Fc-gp120 chimeras were pre-incubated with the inhibitor at RT (unless otherwise noted), then the mixture was added to target cells. In the case of RANTES and TAK-779, cells were also pre-treated in parallel for 30 min at the indicated concentration. For mAb inhibition, we used Fab fragments. Percent inhibition was calculated by the formula 100 - [(*t *- *c*)/(*m *- *c*) × 100], where *t *represents the signal for the test sample, *c *represents the background signal in the absence of Fc-gp120, and *m *represents the signal obtained for Fc-gp120 in the absence of inhibitor.

### Neutralization and attachment assays

In a series of attachment and neutralization assays, we employed JR-CSF pseudovirus as well as live virus to investigate the activity of DC-SIGN and 2G12. In method i) pseudoviruses were used, produced as described previously by transfection of 293T cells with pNL4-3.Luc.R-E- and HIV-1_JR-CSF _Env-expressing plasmids [86,88]. In methods ii) and iii), live viruses harvested after transfection of 293T cells were used [42].

#### i) Standard and post-CD4 neutralization assays

Briefly, virus was incubated with cells for 2 h. Luciferase activity was measured as described previously [88]. Neutralization was measured in two formats with all incubations at 37°C. In the standard neutralization assay format [46], virus was incubated with graded concentrations of inhibitor for 1 h before transferring to CF2Th.CD4.CCR5 cells for a further 2 h incubation, including a 15 minute spinoculation step to increase the assay sensitivity. In the "post-CD4 binding" neutralization format, we preincubated pseudovirions with sCD4 (10 μg/ml) for 15 minutes. Next, the virus-sCD4 cocktail was mixed with graded amounts of mAb for 1 h then allowed to infect Cf2Th.CCR5 cells by spinoculation. All assays were performed at least in duplicate and were repeated for a total of at least 4 replicates to ensure consistency.

#### ii) Neutralization of trans-infection

Briefly, virus corresponding to 10 or 50 ng p24 was incubated with mAbs for 1 hr at RT then added to MDDCs, incubated another 2 h with mixing every 30 min, then the cells were washed 3 times and finally added to TZM-BL cells. After 48 hours co-culture, trans-infection of TZM-BL cells was assayed by a β-gal assay. Results are expressed as % inhibition of infection. As a control, MDDCs were incubated with virus in the absence of nAbs.

#### iii) Inhibition of virus attachment to cells

Inhibition of virus attachment to cells was assessed using a method described previously [42]. Virus (1 ng p24) was pre-incubated (1 h at RT) with b12 or 2G12 at graded concentrations. Virus-mAb mixtures were then added to Namalwa cells (0.25 × 10^6^/500 μl complete RPMI) for 2 hrs at 37°C. Cells were next washed three times in PBS, lysed in triton X-100 and p24 content measured by ELISA. Under these conditions, no internalization occurs, since no cytosolic p24 can be detected upon protease treatment of the target cells, as previously described [19]. Percent inhibition was calculated, as for the FACS analysis.
