# Inhibition of HIV Env binding to cellular receptors by monoclonal antibody 2G12 as probed by Fc-tagged gp120

**DOI:** 10.1186/1742-4690-4-23

**Published:** 2007-03-28

**Authors:** James M Binley, Stacie Ngo-Abdalla, Penny Moore, Michael Bobardt, Udayan Chatterji, Philippe Gallay, Dennis R Burton, Ian A Wilson, John H Elder, Aymeric de Parseval

**Affiliations:** 1 Torrey Pines Institute for Molecular Studies, 3550 General Atomics Court, San Diego CA 92121, USA; 2 Department of Molecular Biology, The Scripps Research Institute, 10550 North Torrey Pines Rd. La Jolla, CA 92037, USA; 3 National Institute for Communicable Diseases, Sandringham, Johannesburg 2131, South Africa; 4 Department of Immunology, The Scripps Research Institute, 10666 North Torrey Pines Rd. La Jolla, CA 92037, USA; 5 Department of Immunology and Molecular Biology, The Scripps Research Institute, 10666 North Torrey Pines Rd. La Jolla, CA 92037, USA; 6 Department of Molecular Biology and The Skaggs Institute for Chemical Biology, The Scripps Research Institute, 10666 North Torrey Pines Rd. La Jolla, CA 92037, USA

After publication of our work [1], we noted that panel C from figure 4 (see Figure 1) was missing. We have now added the corrected figure.

**Figure 1:**
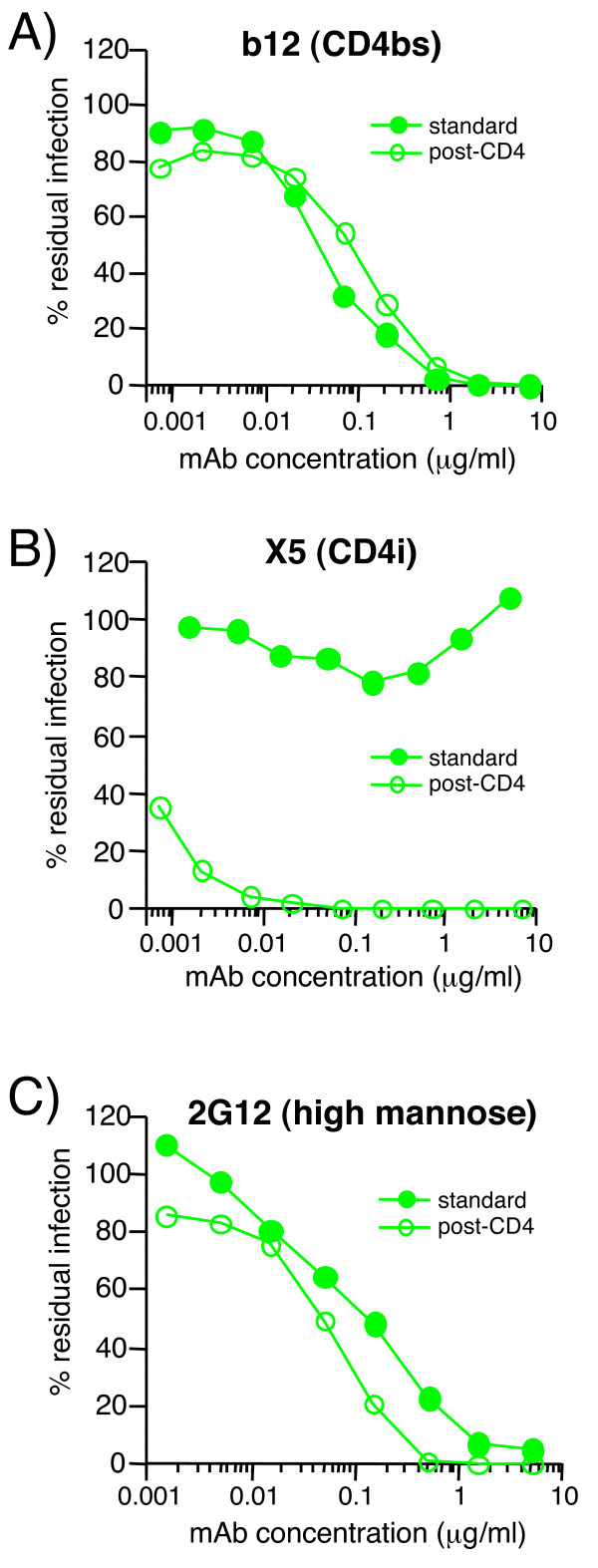
Figure 4. 2G12 neutralizes HIV-1 JR-CSF effectively in a post-CD4 assay format. The neutralization activity of mAbs A) b12, B) X5, and C) 2G12 was assessed in the standard (closed circles) and post-CD4 (open circles) neutralization formats. Results are expressed as % of residual infection, with 100% representing infection in the absence of mAb. Results are representative of two experiments.
